# Ethnobotanical study on wild edible plants used by Dulong people in northwestern Yunnan, China

**DOI:** 10.1186/s13002-022-00501-3

**Published:** 2022-01-21

**Authors:** Zhuo Cheng, Xiaoping Lu, Fengke Lin, Abid Naeem, Chunlin Long

**Affiliations:** 1 Key Laboratory of Ecology and Environment in Minority Areas (Minzu University of China), National Ethnic Affairs Commission of China, Beijing, 100081 China; 2 College of Life and Environmental Sciences, Minzu University of China, Beijing, 100081 China; 3 Key Laboratory of Ethnomedicine (Minzu University of China), Ministry of Education, Beijing, 100081 China; 4 Key Laboratory of Modern Preparation of Traditional Chinese Medicine, Ministry of Education, Jiangxi University of Traditional Chinese Medicine, Nanchang, 330006 China; 5 Kunming Institute of Botany, Chinese Academy of Sciences, Kunming, 650201 China

**Keywords:** Dulong people, Dulongjiang area, Ethnobotany, Wild edible plants, Traditional knowledge

## Abstract

**Background:**

Dulong (Drung people) are one of the ethnic minorities of China, consisting of a small population living in remote and mountainous regions with limited facilities. Over the years, the Dulong have maintained their livelihood by collecting wild medicinal and edible plants. Therefore, through their experience and understanding, they had accumulated sufficient traditional knowledge about local plant resources. Since ancient times, wild edible plants have been essential to the food security of the Dulong people. However, there is almost no comprehensive report available on WEPs consumed by the Dulong people. The objectives of this study were to: (1) make a systematic study of WEPs used by Dulong people, (2) record traditional knowledge related to WEPs, (3) analyze multiple uses of WEPs, and (4) evaluate species with significant cultural significance to Dulong people.

**Methods:**

Ethnobotanical survey including free listing, semi-structured interviews, key informant interviews and participatory observations was conducted in Dulongjiang Township, Gongshan County, Yunnan Province, Southwest China. A total of 127 informants were selected using the snowball method and information about WEPs, including vernacular name, food categories, parts used, mode of consumption, collection season, and other local uses were collected. The RFC and CFSI were calculated to identify the most culturally significant WEPs. One-way analysis of variance was performed to evaluate whether the four reference variables (gender, age, occupation, and education) significantly influenced the number of plant species mentioned by the respondents.

**Results and discussion:**

A total of 148 species of WEPs consumed by the Dulong people belonging to 58 families were collected, including wild vegetables (71), wild fruits (52), staple food substitutes (15), spices (7), nuts (4), tea substitute (2), liquor making materials (3) oils and fats (3), and culinary coagulants (1). WEPs are used in a number of different ways, including as fuelwood, feed, and medicine. Food substitute plants accounted for the majority of the top 27 wild food plants identified by RFC and CFSI. It was observed that farmers have more knowledge of WEPs, and moderate education level informants reported less WEPs used.

**Conclusion:**

The WEPs used by the Dulong people are diverse and abundant in the Dulongjiang region. In the future, WEPs such as *Maianthemum atropurpureum*, *Caryota obtusa*, *Cardiocrinum giganteum*, and *Angiopteris esculenta* with economic potential can be developed to provide a source of income for the residents. More studies of the nutritional value, chemical composition, and biological activities of WEPs are needed. The demands and development of local communities can be realized under the premise of protecting WEPs and the associated traditional knowledge. More attention should be paid to the value of WEP and underutilized plants during future rural development.

**Supplementary Information:**

The online version contains supplementary material available at 10.1186/s13002-022-00501-3.

## Background

China is classified among the countries with the richest biodiversity of plants in the world and has a wide variety of wild edible plants (WEPs) with abundant reserves and a wide distribution [1]. WEPs refer to species that are not artificially planted and domesticated but are collected from the natural environment and used as food sources [2–4]. The collection and consumption of WEPs are an important part of livelihood strategies throughout the world. Furthermore, considered an integral part of local culture, WEPs satisfy the food requirement of different communities [5, 6]. WEPs can not only be used to fill the food gap during droughts or resource scarcity, but also play an important role in maintaining the livelihood security of many people in developing countries and balancing the nutritional value of the diet [7–10]. WEPs are rich in nutrients and have high nutritional value. They can be used as a primary food source by residents and as a food supplement for non-local residents, which guarantees food security in poor communities [10]. WEPs can also be collected in large quantities for processing and sales. Furthermore, it is also one of the main sources of income for residents in poor communities and plays an essential role in helping communities eliminate poverty [11]. They can also serve as a source of domesticated species and provide valuable genetic resources for the development of new crops through hybrid screening [12, 13].

With the development of the social economy and the modernization of agricultural science and technology, human utilization of WEPs has never decreased [14]. Collecting and eating WEPs has become a way of life for modern people, which not only enriches the culture of modern diet, but also satisfies the requirements of a green and nutritious diet [15]. It is estimated that there are about 300,000–500,000 plant species on the planet, of which 30,000 are considered edible, and only 7000 are planted or collected as food. Currently, only 20 crops provide 90% of the world’s food demand [16, 17]. On the one hand, large-scale cultivation of a limited number of crops, coupled with the industrial revolution, lifestyle changes, and lack of contact with nature, has led to the underutilization of WEPs [18]. However, due to the loss of traditional culture and the conversion of forest ecosystems to other types of land use types has also resulted in the loss of traditional knowledge which may be lost completely. The role of WEPs in developing countries has been largely neglected and underestimated [19]. To adapt to continuous population growth and global climate change, diverse food plants are needed to ensure a safe and resilient food supply [20]. In-depth investigations of WEPs and their related traditional knowledge recording are important, (1) to promote the conservation and sustainable use of WEPs, (2) to enable future generations to acquire traditional knowledge associated with WEPs, (3) to help economically backward and low-living areas to uplift themselves out of poverty and food insecurity, and (4) to get germplasm resources with better quality.

Currently, conducting ethnobotanical surveys of wild edible plant resources has attracted the interest of many ethnobotanists and has become the focus of research [21–24]. There are many studies on WEPs in China, mainly focused on the use of plants by ethnic minorities, such as Naxi, Hani, Mongolian, Tibetan, Yi, etc. [3, 6, 25–30]. These studies played an essential role in protecting traditional knowledge and the sustainable use of WEPs and finding the most widely consumed varieties and analyzing their nutritional value [8, 31]. The results of the nutritional analysis will provide clues for the search for excellent germplasm resources, help ensure the diversity of the diet and achieve food security [8, 32, 33].

Drung, or Dulong in Chinese *pinyin*, one of the smallest ethnic groups in China and containing only 6930 people, is mainly concentrated in the Dulongjiang area of northwest Yunnan. Dulong people live near water and choose forests as habitats. In the long process of interaction with the living environment, many WEPs were consumed, and traditional ecological knowledge about them has been accumulated due to unique topography, rainy stereoscopic weather, closed traffic conditions, and abundant natural resources [34–41].

In the past, there have been only sporadic reports on the research of WEPs of the Dulong people, and no comprehensive research and quantitative research has been conducted [34, 41]. From slash-and-burn cultivation to poverty alleviation for the entire tribe, it seems that the traditional knowledge associated with the WEPs of Dulong people will be impacted or even lost. So, it is very necessary to study WEPs from the Dulong ethnic group. The purposes of this study were to (1) conduct a comprehensive study of WEPs used by Dulong people, (2) record the traditional knowledge associated with WEPs, and (3) identify species of important cultural significance to Dulong people.

## Methods

### Study area

**Fig. 1:**
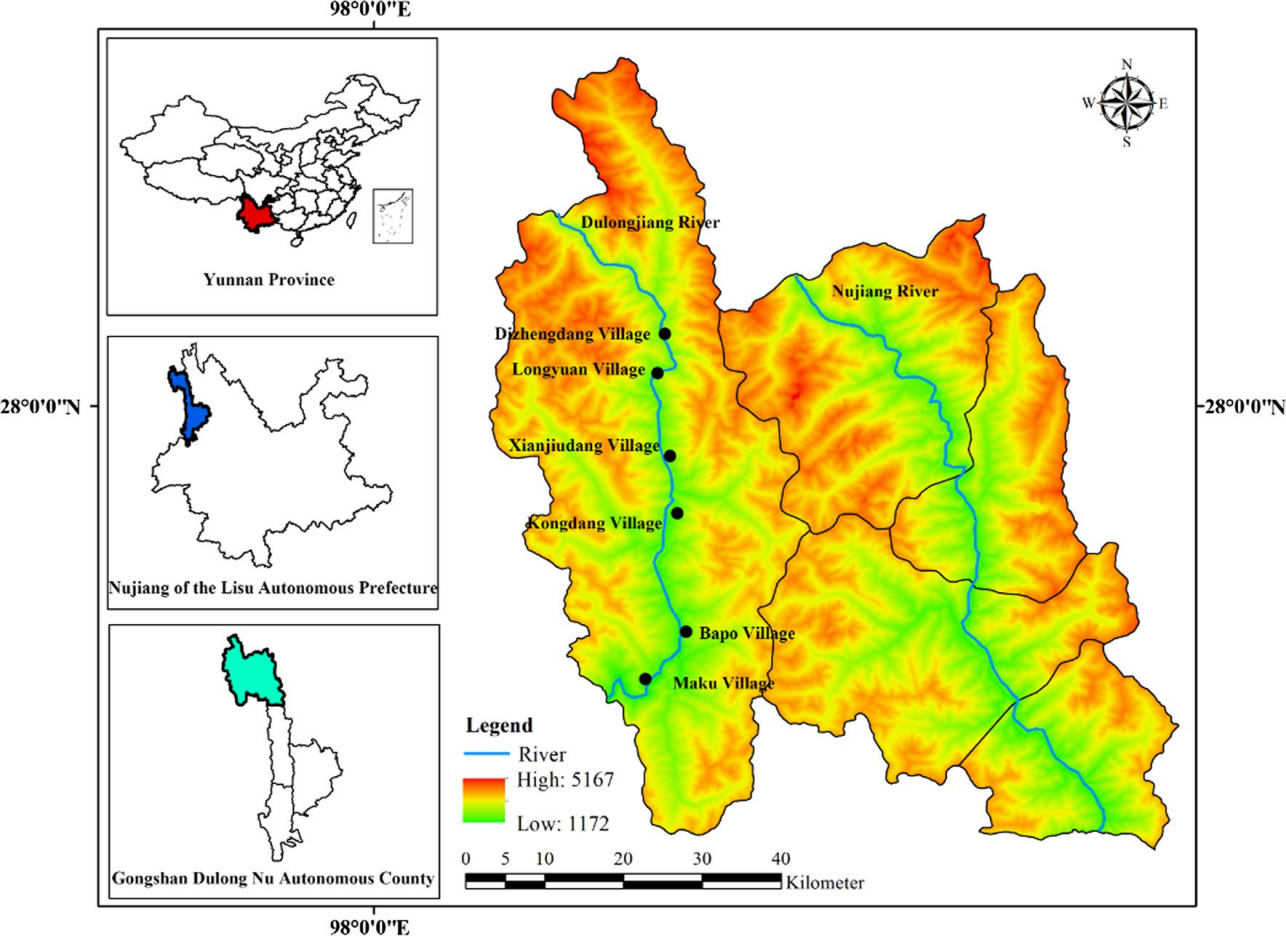
Sketch map of the study area

As the only known settlement of the Dulong in China, Dulongjiang Township is located west of Gongshan Dulong and Nu Autonomous County, Nujiang Lisu Autonomous Prefecture, Yunnan Province, China (Fig. 1). Dulongjiang Township (27° 40ʹ to 28° 50ʹ N, and 97° 45ʹ to the 98° 30ʹ E) is adjacent to Bingzhongluo and Cikai townships in the east, Chayu County of the Tibetan Autonomous Region to the north, and the Kachin State of Myanmar in the west and south [40]. Dulongjiang Township belongs to the typical alpine gorge landform with a large altitude gradient from 1172 to 3400 m. The Dulongjiang region has historically been isolated for a very long time, not being able to communicate with the outside world from December to June of the following year. It remains the most remote, poor, backward and closed region in China. The Dulongjiang area receives an abundance of rainfall (average rainfall of 3672 mm per year), and it is one of the regions with the highest rainfall in China [42]. Dulongjiang Township is an important part of the Gaoligongshan National Nature Reserve and the Three Rivers Parallel World Natural Heritage Site. It still preserves dense virgin forests and is one of the places that have the highest level of biodiversity in China, with 275 species in 41 families of ferns and 2003 species in 158 families of spermatophytes [36, 37, 39]. The diversity of sperm per square kilometer in Dulongjiang is 1.09, which is much higher than that of the autonomous prefecture of Xishuangbanna Dai Autonomous Prefecture (0.19), a very rich area in biodiversity [43]. Dulong ethnic group is one of 55 minority ethnic groups in China, and it has the smallest population in Yunnan province. The Dulong people are considered to be the last seasonal foragers in China [44–46]. The number of Dulong people in Dulongjiang Township represents 99% of the total population (with 6930 people only). Most people speak the Dulong language and only young people can speak Mandarin Chinese. These locals have very low income, with the average annual income of 596 RMB in 2005 and 6122 RMB in 2018, which is lower than the national average level [47].

### Field survey and data collection

From local chronicles, maps and flora, a preliminary understanding was established, including topography, climatic conditions, and vegetation of the Dulongjiang area, history, customs, religious beliefs, and social culture of the Dulong people, before the ethnobotanical investigation, which helped to choose suitable sites and times for the survey. Ethnobotanical surveys were carried out three times in Dulongjiang Township from August 2019 to September 2020, and the entire study covered six villages in Dulongjiang Township (Fig. 1). The data of latitude, longitude and elevation of the six villages are listed in Table 1.

**Table 1:** Study site locations and demographic characteristics of respondents

Village	Gender		Age				Education level				Occupation				Location		
	Male	Female	≤ 19	20–39	40–59	≥ 60	Illiterate	Primary	Secondary	High school and above	Farming	Salary work	Trading	Student	Latitude (north)	Longitude (east)	Distance to the Township (km)
Dizhengdang	11	8	2	5	7	5	6	3	7	3	14	2	2	1	27° 44′ 9ʺ	98° 20′ 58ʺ	Far (23.44)
Longyuan	15	8	0	15	6	2	3	5	13	2	19	4	0	0	27° 52′ 18ʺ	98° 20′ 25ʺ	Far (16.75)
Xianjiudang	14	8	3	8	6	5	2	10	5	5	13	6	0	3	28° 4′ 35ʺ	98° 19′ 33ʺ	Near (7.37)
Kongdang	11	10	0	9	7	5	6	5	7	3	16	5	0	0	28° 0′ 51ʺ	98° 18′ 48ʺ	Near (0)
Bapo	12	10	1	9	9	3	4	2	11	5	14	5	1	2	27° 55′ 9ʺ	98° 20′ 59ʺ	Far (15.07)
Maku	12	8	2	13	4	1	5	2	12	1	12	6	0	2	27° 41′ 18ʺ	98° 17′ 53ʺ	Far (21.36)
Total	75	52	8	59	39	21	26	27	55	19	88	28	3	8			

Field surveys included free listing, semi-structured interviews, key informant interviews and participatory observation, and a total of 127 informants were selected using the snowballing method. During the field investigation, every informant was invited to list all wild plants that are still regularly used or have been used in the past. The interviews consisted of two parts, the first part was about the basic situation of the informants (ethnicity, age, education, occupation), and the other part included questions related to recording detailed information on WEPs, including the local name, availability, use part, processing method, frequency of use, mouthfeel, whether it is used as a medical diet, months of collection, and other uses. Data were used for quantitative analysis are listed in Table 2.

**Table 2:** Questions used for semi-structured interviews

No.	Questions
(1)	What wild plants have you eaten?
(2)	What is the local name of the plant?
(3)	Where do you collect the plant?
(4)	Which part of the plant do you eat?
(5)	How do you process the plant?
(6)	What is the utilization frequency of this plant?
(7)	How does this plant taste?
(8)	Can this plant be used as medicinal food?
(9)	Is there any other use for this plant?
(10)	When is this plant collected?

For the identification of plants, the voucher specimens were studied and compared with reference books (*Flora Rpublicae Popularis Sinicae*, *Flora of China*, *Flora of Yunnan*, *Flora of Dulongjiang Region*) and electronic online resources (http://www.iplant.cn/ and http://www.theplantlist.org/). The nomenclature of all vascular plants follows *Flora of China*, Prof. Chunlin Long identified all plant species, and the voucher specimens were deposited in the herbarium of the ethnobotany laboratory of the College of Life and Environmental Sciences, Minzu University of China, in Beijing.

### Demographic characteristics of the respondents

A total of 127 Dulong respondents were selected (19 from Dizhengdang, 23 from Longyuan, 22 from Xianjiudang, 21 from Kongdang, 22 from Bapo, and 20 from Maku). And 75 of the respondents were male, and 52 were female. The age of the respondents ranges from 9 to 106, and most of the people were in the age group of 20 to 39. The overall education level was poor and young people had a higher education level than the elders. The occupations of the informants included farmers, wage workers, traders, and students, among which the farmers account for the majority (Table 1).

### Relative frequency of citation (RFC)

The RFC was used to quantify the frequency of use of certain species, which was determined using the following formula:

$${\text{RFC}} = \frac{FC}{N}$$

FC refers to the number of respondents who mentioned a particular wild edible plant and N represents the number of all respondents participating in the survey.

The RFC values vary from 0 to 1, and the higher the RFC value, the more important and valuable the plant is in the area. The importance of each wild edible plant was indicated by its FC value, which allowed all WEPs mentioned in importance survey to be listed in the order of importance [48, 49].

### Cultural food significance index (CFSI)

The CFSI index considers a wide variety of factors in the evaluation of a specific wild edible plant. CFSI was calculated to evaluate the cultural significance of wild edibles using the formula given by Pieroni [50].

$${\text{CFSI}} = {\text{QI}} \times {\text{AI}} \times {\text{FUI}} \times {\text{PUI}} \times {\text{MFFI}} \times {\text{TSAI}} \times {\text{FMRI}} \times {1}0^{{ - {2}}}$$

The CFSI includes quotation frequency (QI, frequency of quotation index), availability (AI, availability index), typology of the used parts (PUI, parts used index), frequency of use (FUI, frequency of utilization index), kind and a number of food uses (MFFI, multifunctional food use index), taste appreciation (TSAI, taste score appreciation index) and perceived role as food medicine (FMRI, food-medicinal role index). The use of this index allows for exploring potential wild greens, as they exist in different climatic zones [6].

### Jaccard index (JI)

JI can reflect the similarity between samples. We evaluated similarities between our studies with previous ethnobotanical studies carried out in other parts of China, as well as those from neighboring countries.

$${\text{JI}} = \frac{C}{A + B - C} \times 100$$

A is the recorded number in species of the current study area a, B is the documented number in species of another study area b, and C is the number of species common to both areas a and b [51].

### Data processing

For the analysis, four factors (gender, age, education level, and occupation of the respondents) were used as reference variables [52]. One-way analysis of variance (one-way ANOVA) was performed to evaluate whether the four reference variables had a significant impact on the number of plants mentioned by the respondents. All analysis was performed with SPSS (version 20).

## Results

### Diversity of wild edibles

Dulong people use different varieties of WEPs, and 127 informants reported a total of 148 WEPs. Botanical and ethnobotanical information about these plants, including scientific names, vernacular names, families, life forms, food categories, used parts and mode of consumption, collection season, other local uses, specimen numbers, RFC, and CFSI are listed in Table 3.

**Table 3:**
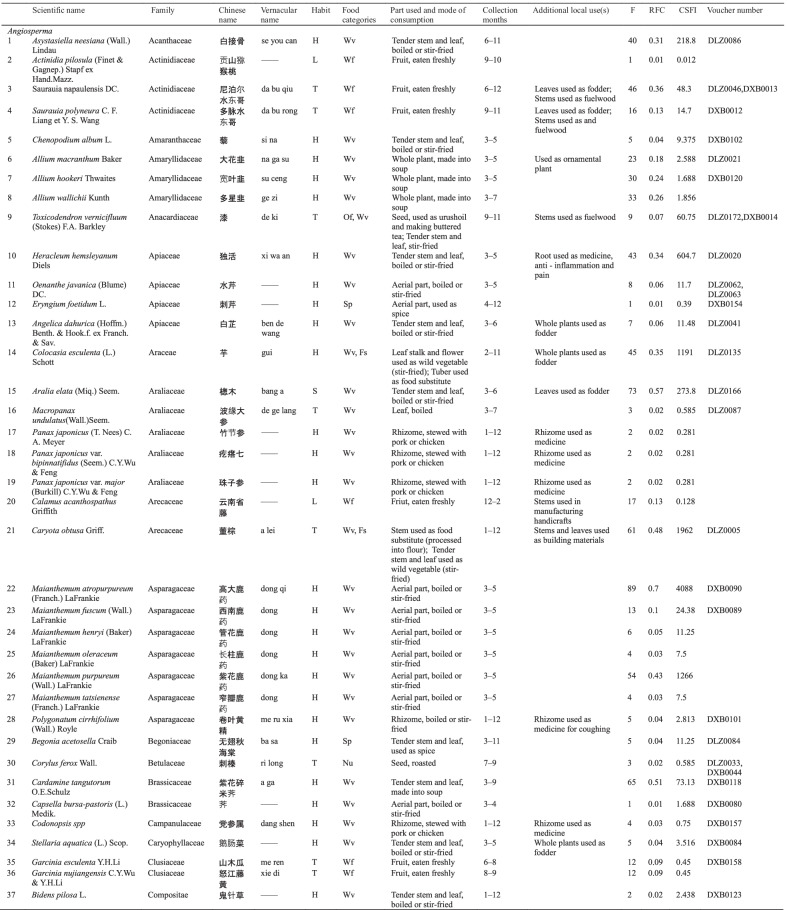

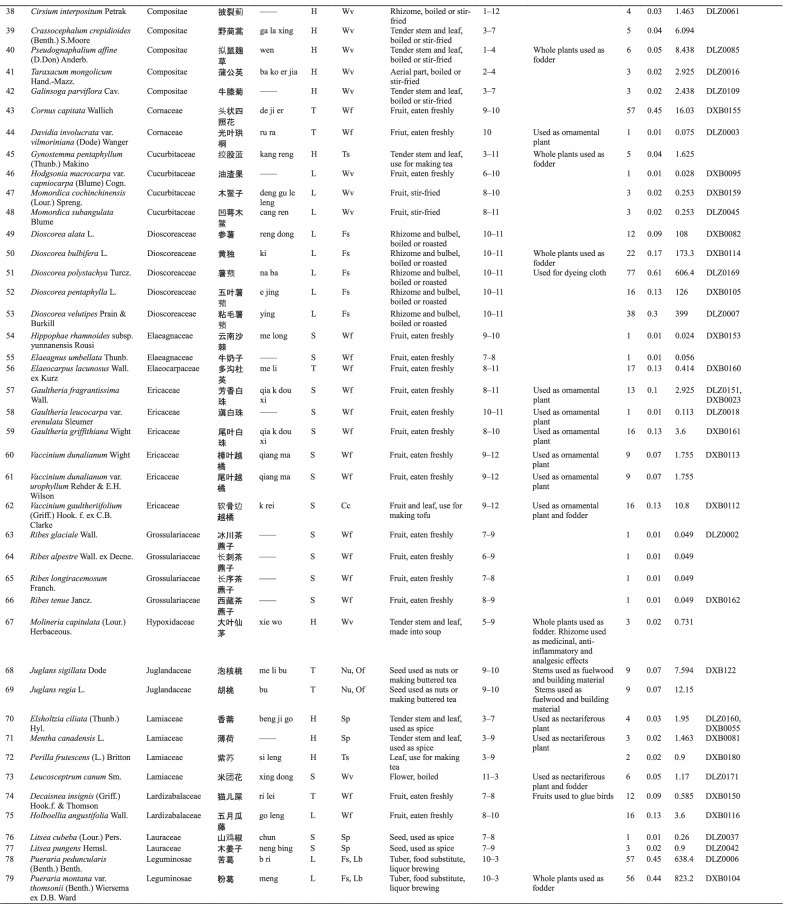

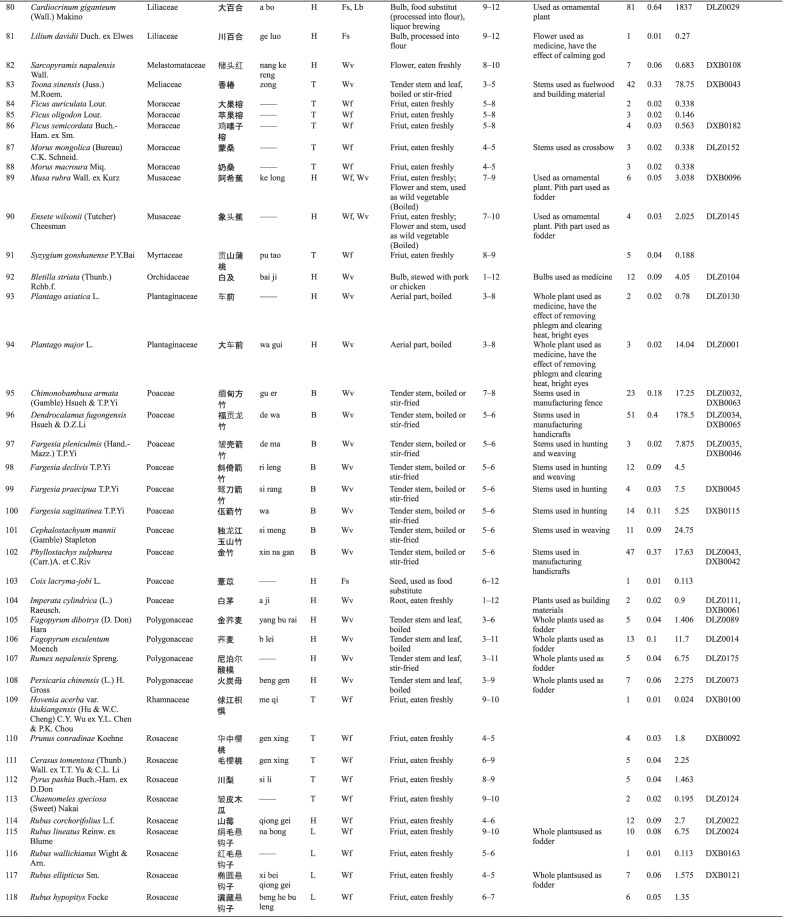

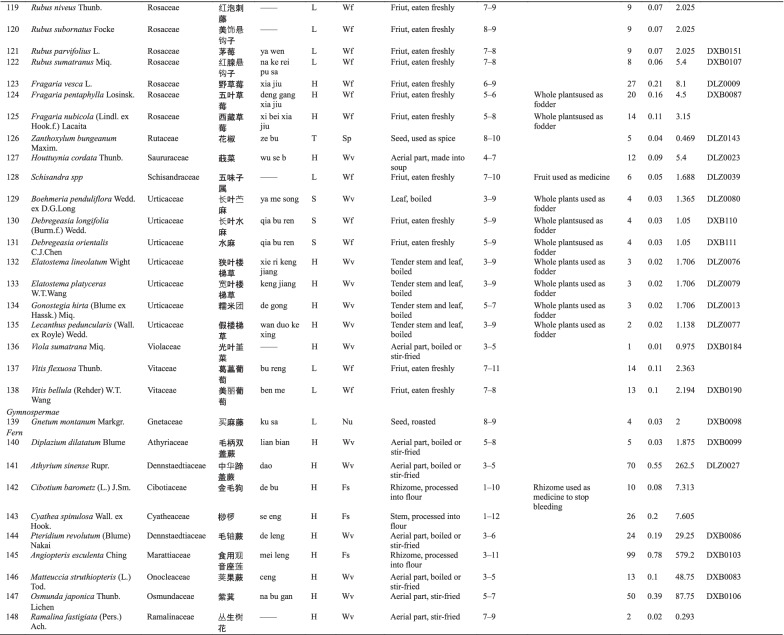
List of wild edible plants used by Drung

**Fig. 2:**
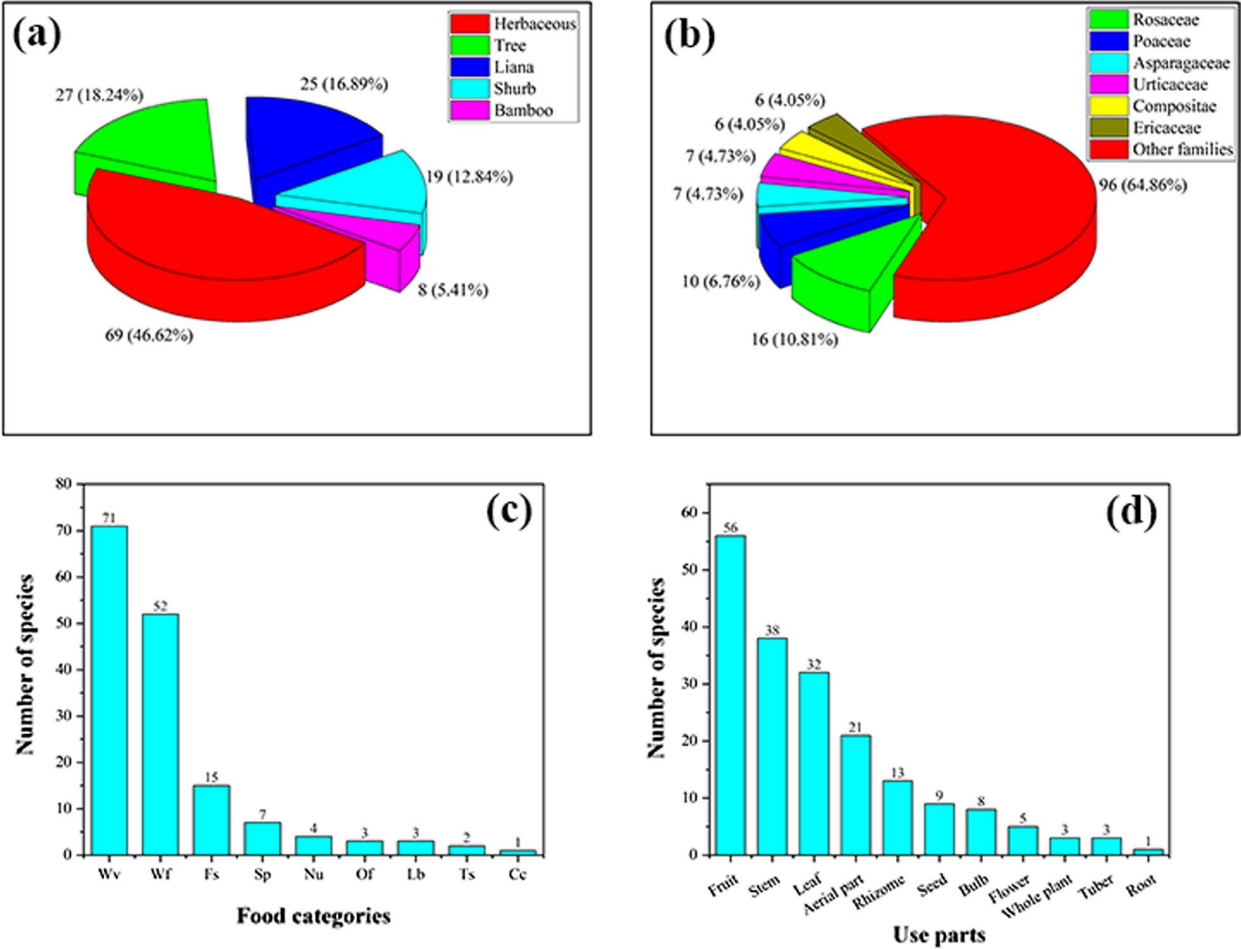
**a** Life forms of WEPs used by Dulong people; **b** Family distribution of WEPs species of angiosperm category; **c** Food categories of WEPs species; **d** Edible parts

The types of WEPs used by Dulong people include angiosperms, gymnosperms, ferns, and lichens. Most of the documented species were angiosperm, with 138 species belonging to 50 families. Rosaceae were found to be the largest family with 16 species, followed by Poaceae with 10 species (Fig. 2b). Fern was the second largest group containing 8 species representing 7 families, while Gymnosperm and Lichen both have one species (one family). The life forms of these WEPs are mostly herbaceous (69) and trees (22) (Fig. 2a).

Many parts of plants are edible, such as stems, leaves, fruits, seeds, flowers, roots, and tubers, among which the most commonly consumed parts are fruits, followed by stems and leaves (Fig. 2d). Wild vegetables and fruits are the two main categories of WEP, and the used parts of wild vegetables include tender stems and leaves, while the parts used parts of wild fruits utilized are fruits.

**Fig. 3:**
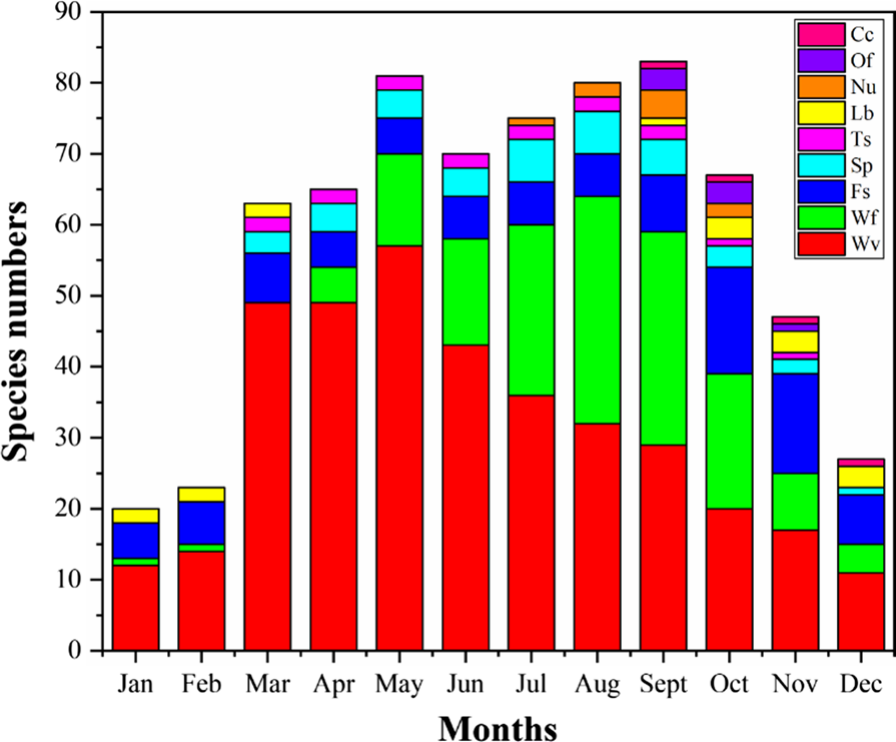
Months of collection for WEPs

WEPs used by Dulong people can be collected throughout the year. The collection time of WEPs depends on the maturation of the parts used, but most of them are collected from March to October. The collection time for wild vegetables is mainly from March to June, and the collection time for wild fruits is from July to October (Fig. 3).

The WEPs used by Dulong people include wild vegetables (Wv), wild fruits (Wf), food substitutes (Fs), nuts (Nu), spices (Sp), tea substitutes (Ts), liquor brewing (Lb), oils and fats (Of) and culinary coagulants (Cc). Among these, wild vegetables are the most commonly used (71), followed by the consumption of wild fruits (52) (Fig. 2c).

#### Wild vegetables

**Fig. 4:**
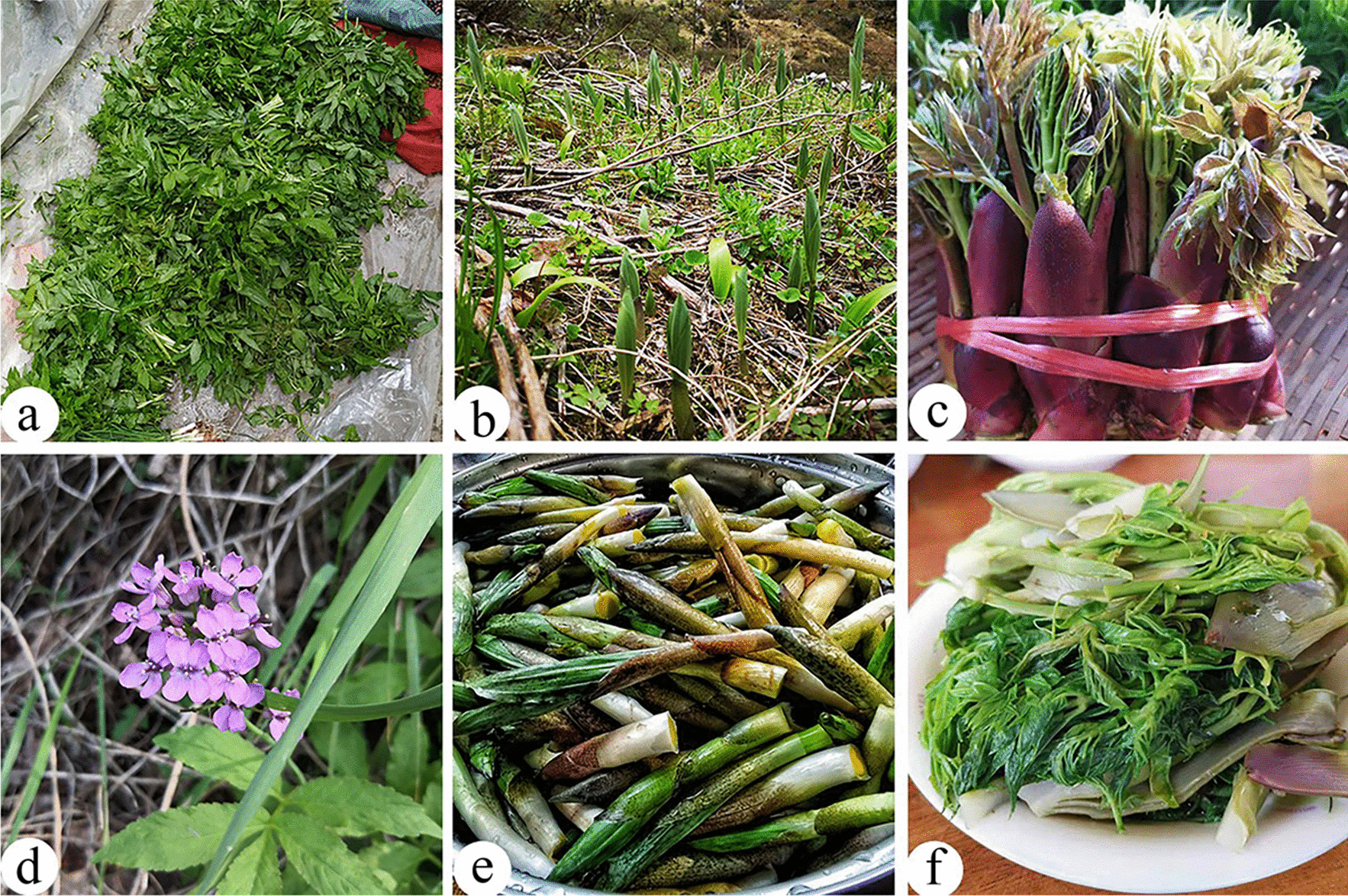
Some wild vegetables in the study area. **a**, **d** *Cardamine tangutorum*; **b**, **e** *Maianthemum atropurpureum*; **c**, **f** *Aralia elata*

Wild vegetables were the most extensively used in the food category with 71 edible species, and the main edible parts of wild vegetables are tender stems and leaves. Wild vegetables are consumed by boiling or stir-frying, or as ingredients in soups or stewed with pork/chicken. The most frequently reported species were *Maianthemum atropurpureum*, *Cardamine tangutorum*, and *Aralia elata*, which are the most popular potherbs in the Dulongjiang region (Fig. 4). These results are in line with previous studies conducted in Sichuan Province and northwest Yunnan Province. These species are also the most popular potherbs of the Yi, Tibetan, and Naxi people [3, 6, 53, 54]. These three species usually grow in the mountains at higher altitudes. Due to the similarity of the leaves of *Maianthemum atropurpureum* with the bamboo leaves, it was called “  ” (Zhu-ye-cai), and Dulong people collected it from March to May annually. The collection time of *Cardamine tangutorum* is much longer than that of *Maianthemum atropurpureum* and can be eaten until September. In addition, *Aralia elata* is cultivated in home gardens. Many studies have shown that these medicinal plants in the diet have high nutritional value and medicinal value [55–58]. There are also other types of abundant potherb sold in local markets of the Dulongjiang region, which have become a source of economic income and have the potential to become important and economically valuable vegetables.

#### Wild fruits

**Fig. 5:**
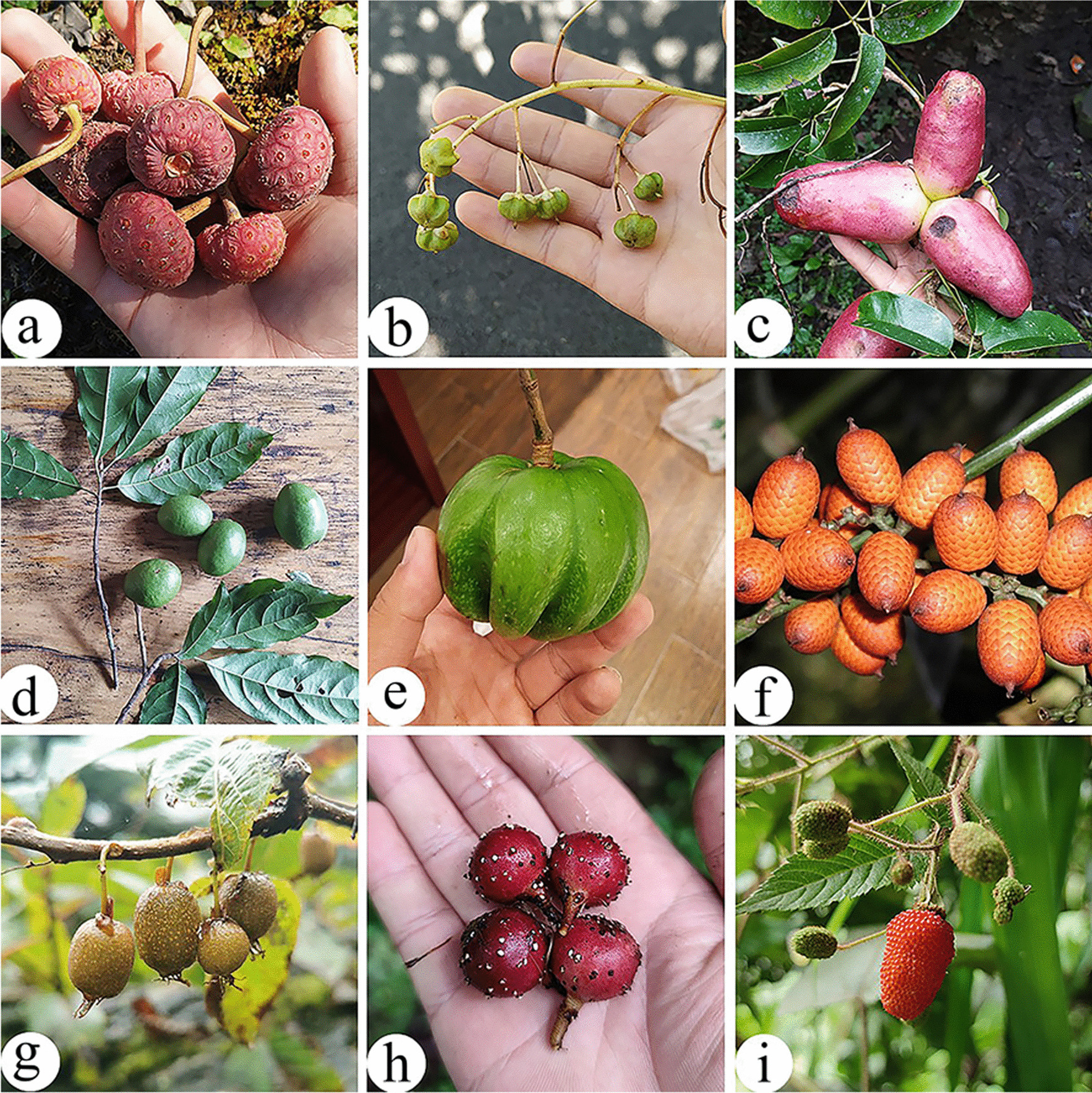
Some wild fruits in the study area. **a** *Cornus capitata*; **b** *Saurauia napaulensis*; **c** *Holboellia angustifolia*; **d** *Elaeocarpus lacunosus*; **e** *Garcinia esculenta*; **f** *Calamus acanthospathus*; **g** *Actinidia pilosula*; **h** *Ficus semicordata*; **I** *Rubus sumatranus*

Wild fruits are the second largest food category of WEPs used by Dulong people, with 52 species. All wild fruits have no market value; they were consumed within families as fresh fruits like snacks. Studies have shown that the nutritional value of wild fruits is higher than that of cultivated fruits [59]. Wild fruits were popular with children as vitamins and minerals supplement, mostly during periods when cultivated fruits were not readily available. The most frequently reported species were *Cornus capitata* and *Saurauia napaulensis* (Fig. 5a, b). *Cornus capitata* is distributed only in Dizhengdang and Longyuan Village, upstream of the Dulongjiang region, while, *Saurauia napaulensis*, also called “  ” (Bi-ti-guo), is distributed throughout the township (Fig. 5c). People in Bapo and Maku Village also eat another “  ” with leaves like brown hairs (*Saurauia griffithii*).

Due to the unique landscape and climate, there are many special wild fruit species (in genera such as *Rubus*, *Gaultheria*, *Ribes*, and *Ficus*) in the Dulongjiang region. These species can be used as new genetic resources for the breeding various fruits, such as *Actinidia pilosula*, *Syzygium gongshanense, and Hovenia acerba* var. *kiukiangensis*, *Elaeocarpus lacunosus*, *Garcinia esculenta*, and *Garcinia nujiangensis* (Fig. 5).

#### Food substitutes

**Fig. 6:**
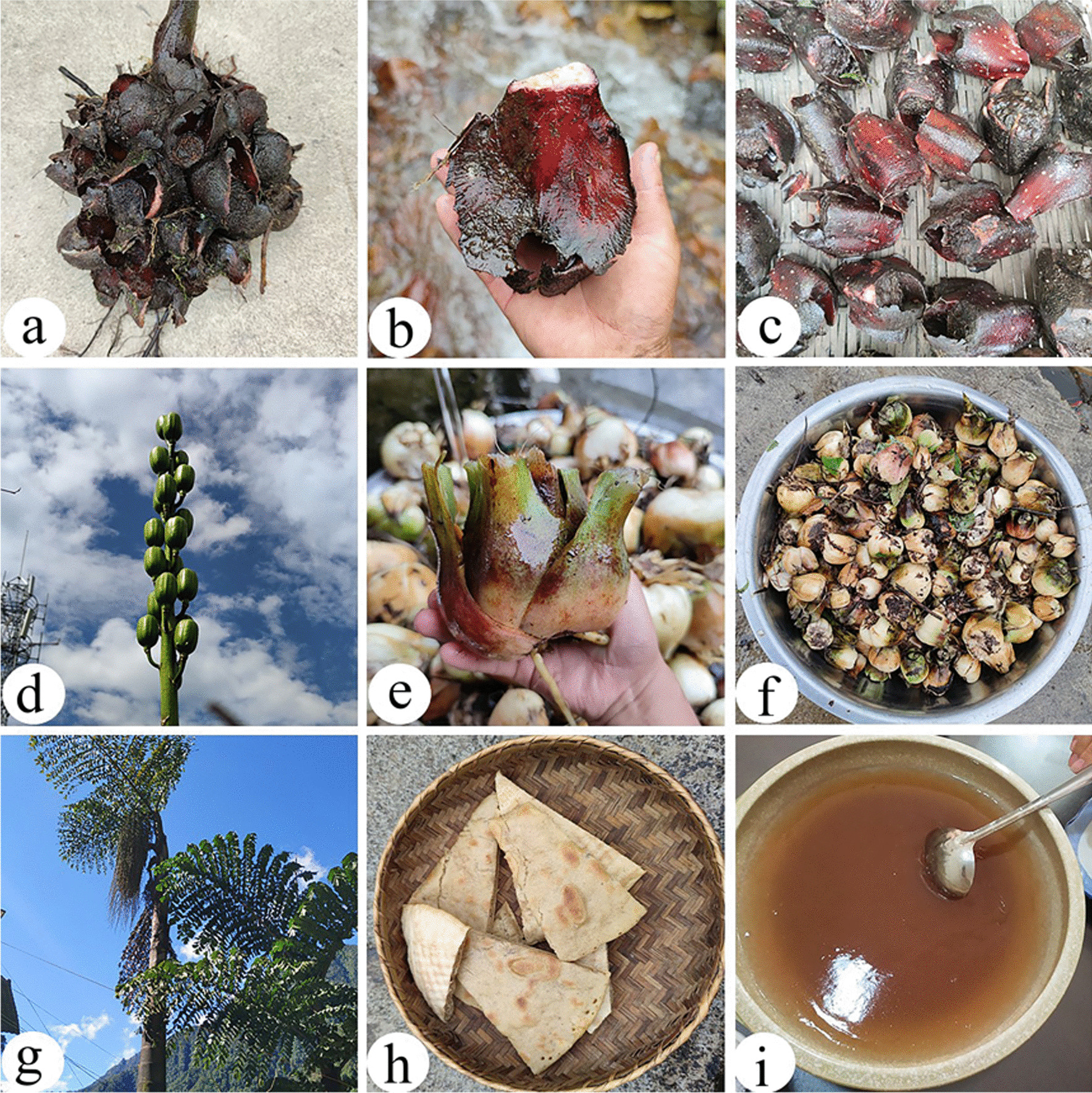
Some staple food substitutes in the study area. **a–c** *Angiopteris esculenta*; **d–f** *Cardiocrinum giganteum*; **g–i** *Caryota obtusa*

Due to the topography and the rainy climate of the mountain valleys in the Dulongjiang region, it is not suitable for crop cultivation of crops [36]. In the past, the Dulong people relied on food substitutes to deal with food shortages. The Dulongjiang region is rich in plant species that can be used as food substitutes, and Dulong people have a unique mode of consumption of food substitutes. Most are processed to starch from tubers, rhizomes, or bulbs and then eaten as Baba (local cake). There are also processed pulp parts, such as *Caryota obtusa* (Fig. 6g, h, i). The most frequently reported species were *Angiopteris esculenta*, *Cardiocrinum giganteum*, *Dioscorea polystachya*, and *Caryota obtusa* (Fig. 6). *Angiopteris esculenta*, an endemic species in NW Yunnan, bears the rhizomes weigh more than 20 kg and the diameter can reach 30–40 cm. In the old days, Dulong people extracted starch from its rhizomes as food [60]. Five *Dioscorea* species were cultivated in home gardens by Dulong people (*Dioscorea alata*, *Dioscorea bulbifera*, *Dioscorea polystachya*, *Dioscorea pentaphylla*, and *Dioscorea velutipes*). The cultivation and management of food substitute plants (such as *Caryota obtusa* and *Cardiocrinum giganteum*) helps Dulong people in surviving the food shortage period.

#### Other categories (spices, beverages, liquor brewing, fats and oils, nuts and culinary coagulant)

**Fig. 7:**
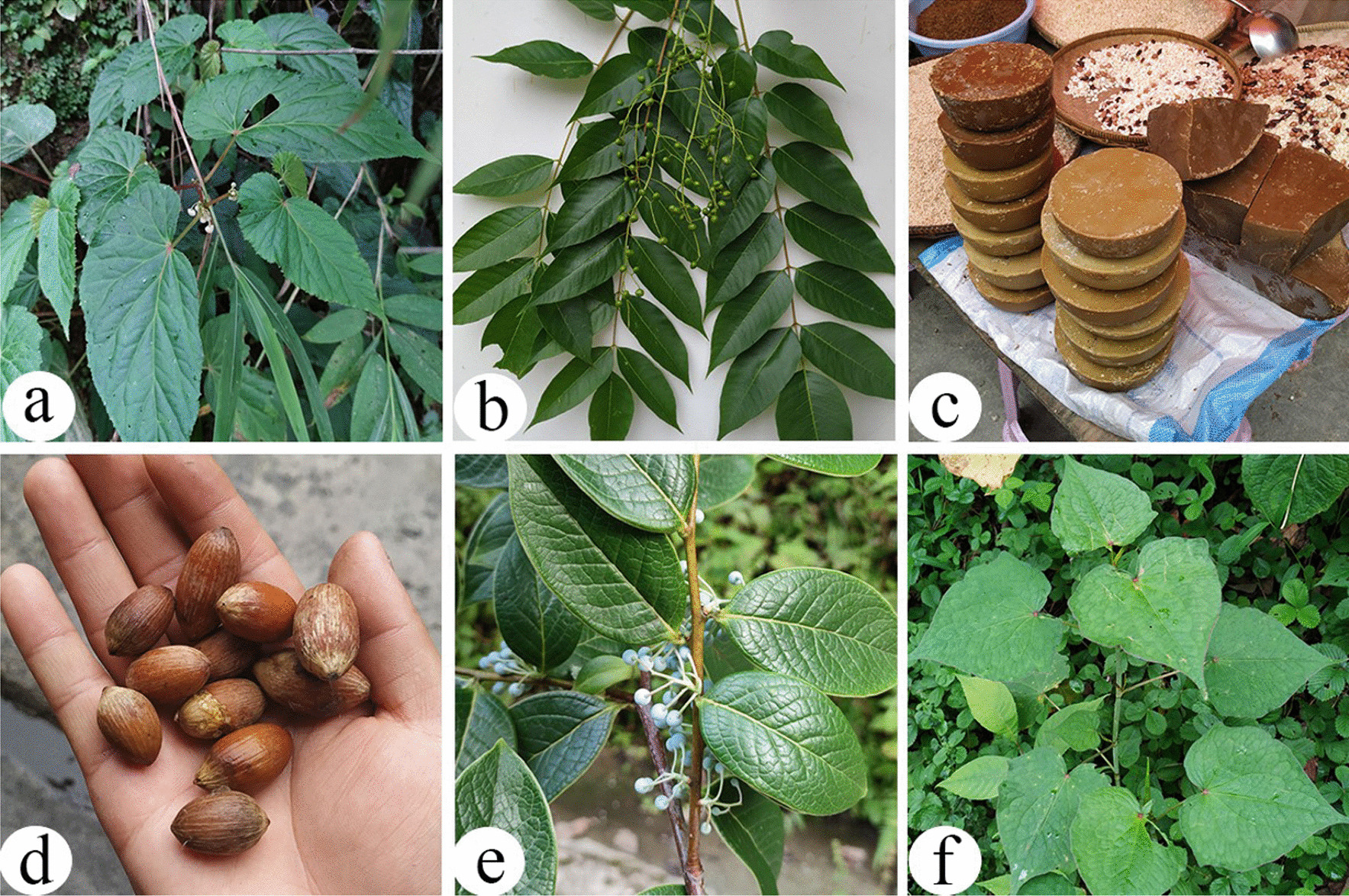
Miscellaneous WEPs in the study area. **a** *Begonia acetosella*; **b**, **c** *Toxicodendron vernicifluum*; **d** *Gnetum montanum*; **e** *Vaccinium gaultheriifolium*; **f** *Fagopyrum dibotrys*

In total, 18 WEPs from other food categories, including spices, beverages, liquor making, oils and fats, nuts, and culinary coagulants, were present. A total of seven edibles were used as spices, and the most frequently reported species were *Begonia acetosella* (Fig. 7a), which is a sour seasoning commonly used by Dulong people and is distributed on the roadsides of both Bapo and Maku villages. Two species (*Gynostemma pentaphyllum* and *Perilla frutescens*) were used as a source of beverages.

The seeds of *Toxicodendron vernicifluum* are used to make oils and fats, which are often used as an excellent tonic during the confinement of pregnant women in Nujiang Prefecture, and comprise approximately 60% unsaturated fatty acids [61]. Locals also pour the melted wax of seed into heated liquor to make wine or tea, which can treat stomach problems [62]. Many people are afraid of lacquer tree, because it contains urushiol, which can easily cause allergies. But few people in the Nujiang area are allergic to lacquer trees or lacquer oil. They even like the lacquer tree very much and have accumulated a lot of traditional knowledge. For example, young leaves of the lacquer tree are used as vegetables, and the trunk of the lacquer tree can be used to make bee barrels and musical instruments, and the management of the lacquer tree based on agroforestry system, etc. [34, 63, 64].

A total of four edibles were used as nuts and the most frequently reported species is *Gnetum montanum* (Fig. 7d). Furthermore, *Vaccinium gaultheriifolium* is a type of culinary coagulant used by the Dulong people to make tofu (Fig. 7e).

A total of three species were used to make wine by Dulong people, including *Pueraria peduncularis*, *Pueraria montana* var. *thomsonii* and *Cardiocrinum giganteum*. *Cardiocrinum giganteum*, also called “  ” (Da-bai-he), is a plant that grows in higher altitudes and barren regions, but is also cultivated by some people (Fig. 6d, e, f). Many excellent agricultural genetic resources are very suitable for cultivation at higher altitudes and barren areas. These high-quality agricultural genetic resources must be protected from extinction.

### Multiple uses of WEPs

**Fig. 8:**
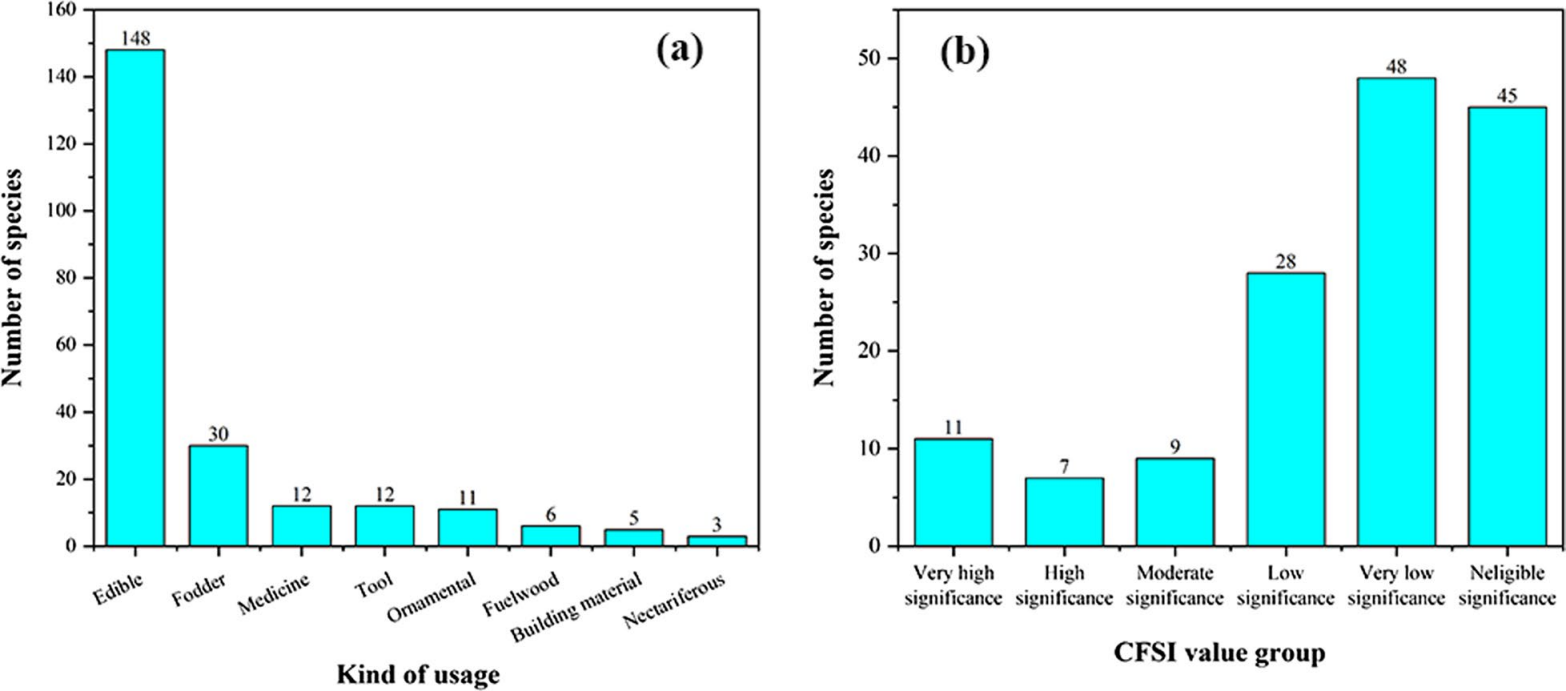
**a** Multiple uses of WEPs; **b** Number of plant species for each CFSI groups

In addition to edible purposes, Dulong people also used 69 plants for other multiple uses (Fig. 8a). Multipurpose uses indicate the importance of wild plant resources to the livelihood and culture of residents [65].

A total of 12 species were found to have medicinal value and are used to treat inflammation, analgesia, hemostasis, cough, and snake bites. These medicinal dietary plants are important for residents to improve health and prevent diseases and can provide raw materials for the development of healthy food [29]. Among medicinal dietary plants, the parts used parts of nine species were underground parts (roots, rhizomes, and bulbs). This pattern of utilization, coupled with overharvesting by local people, has resulted in a massive reduction in plant resources. Therefore, the protection and sustainable use of these medicinal plants should be considered and valued.

Thirty species were used as fodder, of which *Saurauia polyneura*, *Debregeasia orientalis*, and *Rubus lineatus* are excellent fodder for *Bos frontalis* (Gayal) [42]. Dulong women think that *Fagopyrum dibotrys* is good feed for livestock, and they often collect forage plants on the roadside. *Fagopyrum dibotrys* was proven to have a higher protein content than other wild fodders [66]. Eleven species are ornamental plants, most of which belong to Ericaceae (Fig. 6f).

There were twelve plants used to manufacture the tool. The Dulongjiang region is rich in wildlife, and the Dulong people used to survive hunting in the old days. The fruit of *Decaisnea insignis* can be used to stick birds, and *Morus mongolica* was used to make bows, while the stems of four bamboo species were used to make arrows (*Fargesia pleniculmis*, *F. declivis*, *F. praecipua*, and *F. sagittatinea*).

### Evaluation and selection of wild edibles based on RFC and CFSI values

The values of the cultural food significance index (CFSI) varied considerably from one species to another, with a minimum of 0.01 and a maximum of 4088.44 (Additional file 1, Appendix A). When considering the values of this index, according to Pieroni [24], it was possible to classify the plants cited plants into six groups: species with very high significance (CFSI = 300 and above), high significance (CFSI = 100–299), moderate significance (CFSI = 20–99), low significance (CFSI = 5–19), very low significance (CFSI = 1–4) and negligible significance (CFSI < 1). These groups vary in size, with most of them belonging to the very low significance group (48) and negligible group (45) (Fig. 8b).

Twenty-five species of wild edibles [very high group (11), high group (7), and moderate group (9)] were selected using quantitative indices (CFSI). The higher the CFSI value, the more important the role this plant plays in the diet [6]. The corresponding RFC value and ranking of each plant are also listed in Table 4. Furthermore, 20 of 27 wild food plants screened by the two indexes are the same, indicating that the plants screened by the two indexes are very consistent. The ranks of some species based on different indices were different, indicating that different indices assigned diverse importance to various attributes, such as medicinal use and taste appreciation.

**Table 4:** Evaluation of wild edibles using CFSI and RFC index

Species names	Vernacular name	Indices		Ranking	
		CFSI	RFC	CFSI	RFC
*Maianthemum atropurpureum*	dong qi	4088	89	1	2
*Caryota obtusa*	a lei	1961	61	2	8
*Cardiocrinum giganteum*	a bo	1837	81	3	3
*Maianthemum purpureum*	dong ka	1265	54	4	12
*Colocasia esculenta*	gui	1190	45	5	17
*Pueraria montana* var. *thomsonii*	meng	823.2	56	6	11
*Pueraria peduncularis*	b ri	638.4	57	7	10
*Dioscorea polystachya*	na ba	606.4	77	8	4
*Heracleum hemsleyanum*	xi wa an	604.7	43	9	18
*Angiopteris esculenta*	mei leng	579.2	99	10	1
*Dioscorea velutipes*	ying	399.0	38	11	21
*Aralia elata*	bang a	273.8	73	12	5
*Athyrium sinense*	dao	262.5	70	13	6
*Asystasiella neesiana*	se you can	218.8	40	14	20
*Dendrocalamus fugongensis*	de wa	178.5	51	15	13
*Dioscorea bulbifera*	ki	173.3	22	16	29
*Dioscorea pentaphylla*	e jing	126.0	16	17	34
*Dioscorea alata*	reng dong	108.0	12	18	47
*Osmunda japonica*	na bu gan	87.75	50	19	14
*Toona sinensis*	zong	78.75	42	20	19
*Cardamine tangutorum*	a ga	73.13	65	21	7
*Toxicodendron vernicifluum*	de ki	60.75	9	22	56
*Matteuccia struthiopteris*	ceng	48.75	13	23	44
*Saurauia napaulensis*	da bu qiu	48.30	46	24	16
*Pteridium revolutum*	de leng	29.25	24	25	26
*Cephalostachyum mannii*	si meng	24.75	11	26	53
*Maianthemum fuscum*	dong	24.38	13	27	40

Eleven food substitute species were evaluated and selected based on FC and CFSI. In the past, the Dulong people had poor living conditions and often faced a lack of clothing and food, so many plants were used as a substitute for food. These grain substitute plants have been gradually introduced into their home gardens for cultivation in the previous wild state.

*Caryota obtusa* is a secondary protected plant species distributed mainly distributed in its Maku village, and the cultivation and management of it have a stronger position in the development history of the Dulong people. After processing one mature tree, about 100–300 kg of sago starch can be obtained. Therefore, as an important food substitute plant, it helped Dulong people manage the resource shortage food gap during the period of resource shortages and played an important role in maintaining the livelihood security of the Drung and the nutritional value of a balanced diet. During their farm work, Dulong people will remove weeds for *C. obtusa* or cultivate some seedlings near their houses from the mountains. Generally, when there are major events, such as building a house or celebrating a marriage, *C. obtusa* will be planted in the home gardens in case of unexpected needs. To adapt to the harsh environment, a special cultivation and management mode for *C. obtusa* was generated by Dulong people. This traditional knowledge is related not only to the local biophysical environment but also to the traditional culture, which helped to maintain the population of *C. obtusa* and guaranteed regional food security. There are several ecological concepts in the mode of managing and utilizing *C. obtusa* by Dulong people, which provides a reference method for the cultivation and protection of *C. obtusa* resources.

### Comparison of WEPs between Dulong people and other ethnic groups in different areas

To illustrate the homogeneity of WEPs between different places, the JI was used to compare our study with eight previous investigations in China and neighboring countries [3, 6, 7, 67–71]. In total, the JIs of four regions were calculated in China, with Shangri-la emerging as the most similar to our study area with JI = 12.06, followed by the Mêdog County and Lijiang area (JI = 7.21, and 5.25, respectively), while the lowest JI (2.53) was found with the study conducted in Longzi County. The high JI may reflect that the study area is located in the same geological zone, with similar socioeconomic and cultural characteristics. On the other hand, among three neighboring countries (Myanmar, Nepal, and Pakistan), the similarities in these four places were very low (Table 5).

**Table 5:** Jaccard similarity index (JI) for local and neighboring countries

Nationality	Study area	Indices			JI	References
		A	B	C		
*China*						
Tibetan	Shangri-la region	148	168	34	12.06	[3]
Naxi	Lijiang area	148	173	16	5.25	[6]
Lhoba	Longzi County	148	55	5	2.53	[62]
Monpa	Mêdog County	148	194	23	7.21	[63]
*Neighboring countries*						
——	Nepal	148	132	4	1.45	[65]
——	Western Nepal	148	72	15	7.32	[67]
——	Myanmar	148	81	6	2.69	[64]
——	Lesser Himalayas-Pakistan	148	44	2	1.05	[66]

**Fig. 9:**
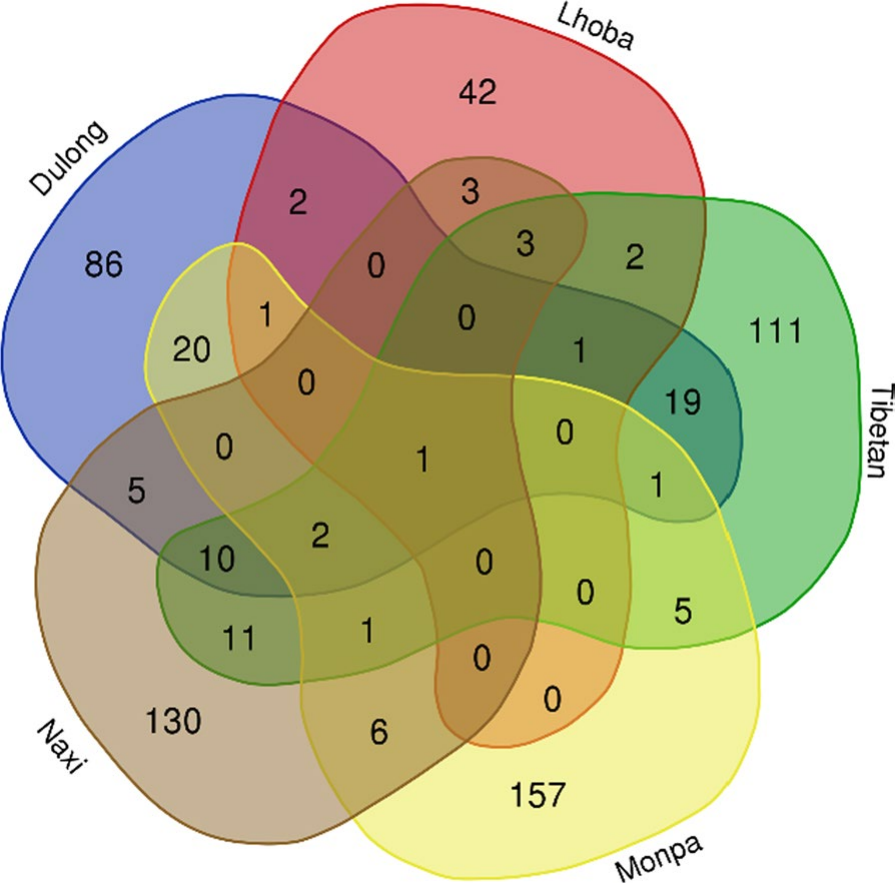
Comparison of WEPs between Dulong and other ethnic groups in China

To explore WEPs differences in the use of WEPs between the Dulong and several other ethnic groups in northwest Yunnan, China, we used a Venn diagram to visualize WEPs used by the five ethnic groups (Dulong, Tibetan, Naxi, Lhoba and Monpa). There is only one plant species, namely *Chenopodium album*, used by all 5 ethnic groups (Fig. 9). There are two species (*Elaeagnus umbellata* and *Cornus capitata*) used by Dulong and other three ethnic groups.

There are 13 species (*Ribes alpestre*, *Rubus ellipticus*, *Rubus niveus*, *Capsella bursa-pastoris*, *Zanthoxylum bungeanum*, *Galinsoga parviflora*, *Taraxacum mongolicum*, *Houttuynia cordata*, *Pyrus pashia*, *Plantago asiatica*, *Mentha canadensis*, *Fagopyrum dibotrys*, and *Debregeasia orientalis*) used by Dulong and other two ethnic groups. Dulong, Tibetan, and Monba showed higher similarities of consuming WEPs, which may be related to their living habits and types of surrounding plants.

## Discussion

### Effects of gender, age, education level, occupation and remoteness on traditional edible plant knowledge

Human factors are critical to the inheritance of wild edible plant knowledge. Age, gender, education level, and occupation of informants are factors commonly considered in research [52, 53]. In this study, there is a significant correlation between the amount of knowledge of wild edible plant knowledge mastered by Dulong people with the occupation and education level of informants. However, there is no significant correlation between those numbers and the gender and age of the respondents (Additional file 1, Appendix B).

Among the 127 respondents, men and women represented 59.06% (75) and 40.94% (52) of the total, respectively. Previous studies have shown that gender is a critical variable that influences the distribution of local knowledge, and women often have more traditional knowledge because they are usually unemployed in rural areas and dedicate themselves to household and subsistence activities [6]. However, there are no significant difference between the male and female groups in the Dulongjiang region. This finding is similar to some previous some research [30, 72]. Khakurel et al. also noted that there was no significant difference between genders in terms of total number of species, while categorically analyzing vegetable and fruit groups, there was a significant difference between genders [71]. Due to the harsh geographical and climatic conditions, women are more responsible for collecting near plants and cooking and are generally unable to participate in filed gathering, and men mostly collect wild plants.

The average number of species is 6.1 mentioned by respondents under 19 years of age, 18.0 by the respondents between 20 and 39, 22.9 by respondents between 40 and 59, and 29.9 by respondents over 60. There is no homogeneity of variance in the one-way analysis of variance (< 0.05) (Table 6). A previous study of Naxi people in northwest Yunnan shows that older people play an important role in maintaining knowledge of WEPs [6].

**Table 6:** One-way analysis of variance

Characters	Total number of respondents	Average WEPs no. mentions	df	Homogeneity of variance test	*P* value
*Gender*			1	0.977	0.055
Male	75	21.9			
Female	52	19.0			
*Age range*			3	0.041	–
≤ 19	8	6.10			
20–39	59	18.0			
40–59	39	22.9			
≥ 60	21	29.9			
*Education level*			3	0.269	0.006
Illiterate	26	25.1			
Primary	27	17.9			
Secondary	55	21.0			
high school and above	19	18.1			
*Occupation*			1	0.741	0.000
Farming	88	22.6			
Other occupations	39	16.5			
*Distance to township*					
Near village	43	18.7	3	0.237	0.053
Far village	84	21.8			

**Fig. 10:**
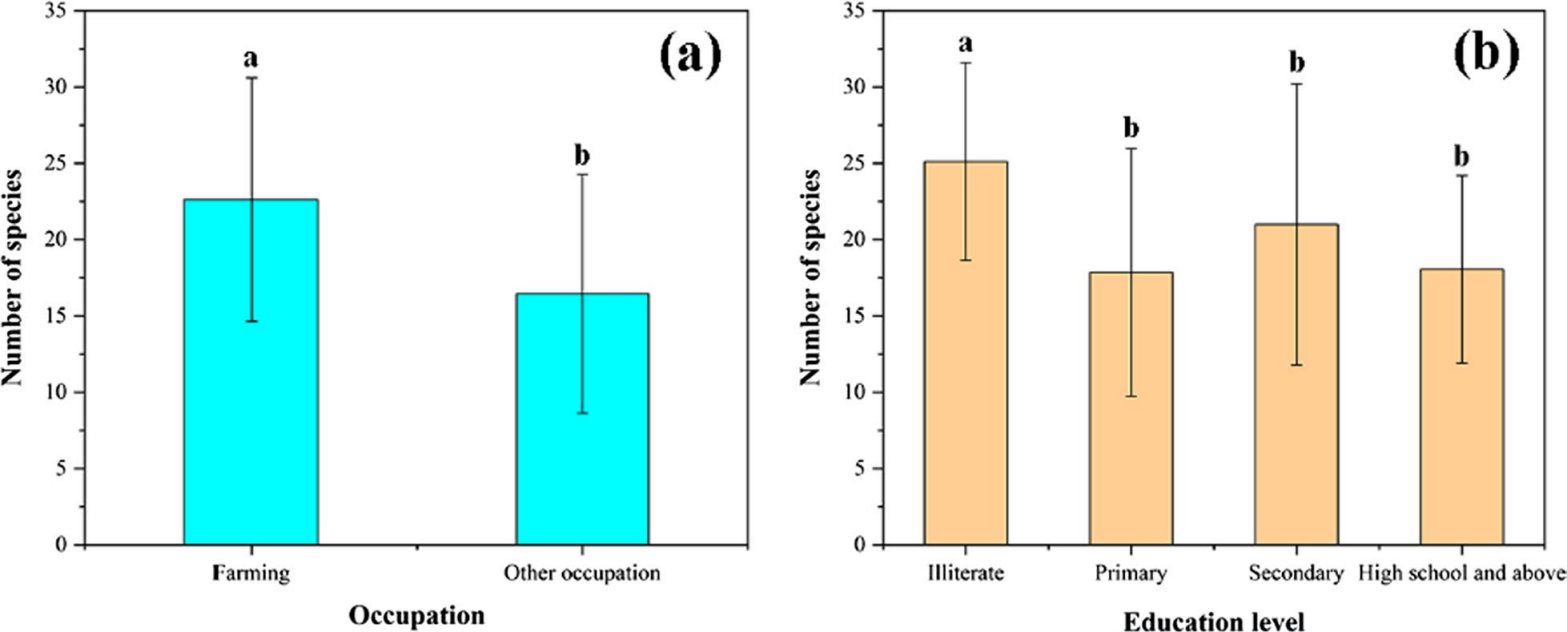
Pairwise comparisons of age and education level. **a** Occupation; **b** Education level

From the education point of view, the uneducated group had the highest knowledge of WEPs (average number of species mentioned is 25.1) and had more traditional knowledge than other groups (*p* < 0.05, compared to other groups). Maybe it is because uneducated people are more dependent on agricultural activities, while educated people generally choose non-agricultural jobs. With the development of the economy and the improvement of living conditions, many people are willing to choose non-agricultural activities, which is one of the reasons for the loss of knowledge retention and transmission of WEPs (Fig. 10b).

From a occupation perspective, the number of WEPs in the hands of agricultural workers represents the most considerable portion (average 22.6), 14.5 by respondents with other occupations. There is a significant correlation with other occupations (Fig. 10a). An important reason is that the Dulongjiang region has always been a remote and backward area with inconvenient transportation, so farmers have been involved in agricultural work and are very familiar with local WEPs. Farmers retain more knowledge of WEPs than people engaged in other occupations (salary work, trade, and students) in the Dulongjiang region.

For the remoteness of the village, the average number of species mentioned by the respondents in the vicinity of the village is 18.7 and 21.8 by the respondents in the far village. However, there are no significant differences between the far village and the nearby village in the Dulongjiang region (Table 6).

### Opportunities and challenges of WEPs

The Dulong people have invaluable knowledge of WEPs, the use of which is generated under a specific cultural and ecological background. Due to the unique topography, climatic conditions, and extremely high biodiversity of the Dulongjiang region, it has generated abundant wild edible plant resources, which is its ecological foundation [36]. Although Dulong people is an ethical group whose main livelihood is collecting, fishing, hunting, and slash-and-burn; they worship the fire pond culture and have a long history of using WEPs and therefore have accumulated a lot of traditional knowledge of WEPs for a long time, which is its cultural foundation.

Recently, with the improvement of living standards, the requirements for dietary balance and variety of foods have gradually increased, and wild food plants have created unprecedented opportunities, especially in the domestication of WEPs, the selection of excellent varieties, the development of WEPs with economic potential and WEPs for both medicine and food. Some similarities exist between medicine and food, and many plants are both edible and medicinal. Plants in local food cultures are inseparable from traditional therapeutic systems [29]. These medicinal dietary plants play an important role in ensuring food and medical safety. Therefore, these plants serve as an established source of income for Dulong people [35].

At the same time, WEPs also face many challenges with economic development and improvements in transportation. Many studies from various regions have found that sociocultural factors are the main drivers of reduced consumption of WEPs [73, 74]. Other studies pointed out that the main drivers of decreased abundance are perceived to be land-use change and direct exploitation of WEPs. These changes have potential negative implications on food systems from local to global scales [75]. The resources of WEPs are constantly threatened by various natural factors and human activities. Furthermore, global climate change leads to various extreme weather events, for example, thunderstorms, mudslides, and flash floods, which significantly contribute to large-scale plant deaths. In addition, various human activities (single-crop cultivation, habitat destruction, excessive harvesting, overgrazing, etc.) also pose a considerable threat to wild plant resources. For example, planting large amounts of *Amomum tsaoko* in the Dulongjiang region to increase income and promote economic development significantly impacts the local understory vegetation. Moreover, not only are plant resources threatened, but traditional knowledge related to these resources is also gradually being lost (Additional file 1).

It seems to have happened within one overnight that Dulong people completed the transition from traditional livelihoods of gathering, fishing, and hunting to the poverty alleviation of entire tribes, and the traditional knowledge of WEPs is bound to be impacted. With local residents' incomes rising, many people are reluctant to collect WEPs, and the younger generation is not very interested in them, which is the main reason for the loss of traditional knowledge of WEPs.

## Conclusion

In summary, 148 WEPs and associated traditional knowledge used by Dulong people were recorded. Multiple uses of these WEPs were analyzed and the most culturally significant WEPs of the Dulong people were identified by quantitative methods. In the future, wild vegetables and fruits with economic potential can be developed to be a source of income for residents. The excellent traits of WEPs can be preserved and exploited by cross-breeding new varieties. More detailed analysis of the nutritional value, chemical composition, and biological activities of WEPs is expected to be performed. The needs of Dulong people and the development of the local community can be realized under the premise of protecting WEPs and related traditional knowledge.

## Supplementary Information

**Additional file 1.** **Appendix A:** Table 1 CFSI data. **Appendix B:** Table 2 One-way ANOVA data.

## Data Availability

All data generated or analyzed during this study was included in this published article (along with the supplementary files).
